# Meetings of Societies

**Published:** 1950-04

**Authors:** 


### Meetings of Societies

---

### British Medical Guild

A meeting to which all members of the profession in Bristol were invited was held at the University on February 15th, 1950, with the object of setting up the local organization of The British Medical Guild. After electing Dr. T. Milling as chairman, the meeting considered the composition of the area committee and decided it should consist of sixteen members. These will be representative of all branches of the profession by having:

Three nominated by the Executive Committee of the Bristol Division of the B.M.A.

Three nominated by the Local Medical Committee.

Three nominated by the Local Consultants' and Specialists' Committee.

One representative of the Public Health members of the area.

Six elected by the meeting, being representative of the local profession.

The following were then elected to fill these places: Drs. B. E. McConnell, H. K. V. Soltau, R. P. Warin, Winifred Nott, J. A. Birrell and W. H. Hayes.

When fully constituted the Committee will meet and elect its officers. Meanwhile, any further information may be obtained from Dr. W. H. Hayes or Dr. H. M. Golding, 10 Barton Hill Road, Bristol 5.

### Devon and Exeter Medico-Chirurgical Society

Clinical Meeting, Thursday, January 5th.

Dr. Gipson and Dr. Daly: **"Diaphragmatic Hernia".**

Mr. Gairdner: **"Five Cases of Carcinoma of the Bladder".**

Dr. Shave and Dr. Seward: **"Chronic Nephritis".**

Dr. Daly: **"Malignant Hypertension".**

At a Clinico-Pathological meeting on March 16th, 1950, Dr. N. S. Alcock presented an unusual case of Hodgkin's disease with lesions confined to the suprarenals, meninges, cord and brain; Mr. F. C. Durbin a case of chondroma of the os calcis following an injury to the heel; Mr. P. G. Scott, Drs. A. V. Daly and Alcock two cases of intracranial abscess, one cerebral, the other cerebellar. The pathological aspects of all four cases, which evoked much lively questioning and comment, were presented by Dr. G. Stewart Smith. The chair was taken by the President of the Society, Dr. Charles Wroth.

### BRISTOL MEDICO-CHIRURGICAL SOCIETY

### INCOME AND EXPENDITURE ACCOUNT for the year ended 30th September, 1949

| EXPENDITURE | £ | s. | d. | INCOME | £ | s. | d. |
|---|---|---|---|---|---|---|---|
| To Subscriptions to *Medico-Chir. Journal* .. .. .. .. .. .. | 380 | 0 | 0 | By Members Subscriptions .. .. | 599 | 0 | 6 |
| ,, Grant to Medical Library .. | 25 | 0 | 0 | ,, Interest on Bank Deposit Account .. .. .. .. .. | | 14 | 2 |
| ,, Librarian, Honorarium .. .. | 15 | 0 | 0 | ,, Interest on £500 4 per cent. Consol. Stock, *less* Tax .. | 11 | 0 | 0 |
| ,, Secretary, Honorarium .. .. | 10 | 0 | 0 | ,, Deficiency for the Year* .. .. | 61 | 6 | 2 |
| ,, Audit Fee .. .. .. .. .. .. .. | 8 | 8 | 0 | | | | |
| ,, Printing and Stationery* .. .. | 88 | 14 | 2 | | | | |
| ,, Lewis's Medical and Scientific Library .. .. .. .. .. | 6 | 18 | 5 | | | | |
| ,, Catering .. .. .. .. .. .. | 104 | 15 | 0 | | | | |
| ,, Other Expenses *re* Meetings .. | 15 | 1 | 2 | | | | |
| ,, Postages and Sundries .. .. | 18 | 4 | 1 | | | | |
| | *£672 | 0 | 10 | | *£672 | 0 | 10 |

### BALANCE SHEET as at 30th September, 1949

| LIABILITIES | £ | s. | d. | £ | s. | d. | ASSETS | £ | d. | s. | £ | s. | d. |
|---|---|---|---|---|---|---|---|---|---|---|---|---|---|
| Sundry Creditors .. .. .. .. .. | | | | 107 | 14 | 0 | Cash at Bank: | | | | | | |
| Subscriptions paid in advance .. | | | | 7 | 18 | 6 | Current Account | 47 | 9 | 6 | | | |
| Cash due to the *Journal** .. .. | | | | 202 | 0 | 0 | Deposit Account | 92 | 2 | 8 | | | |
| Accumulated Fund as at 30th September, 1948 | 594 | 2 | 4 | | | | | | | | 139 | 12 | 2 |
| *Less* Deficiency for the year* .. .. .. .. | 61 | 6 | 2 | | | | Petty Cash in Hand .. .. .. | | | | 2 | 18 | 0 |
| | | | | 532 | 16 | 2 | £500 4 per cent. Consol. Stock at Cost | | | | 437 | 9 | 0 |
| | | | | | | | Arrears of Subscriptions .. .. | | | | 270 | 9 | 6 |
| | | | | £850 | 8 | 8 | | | | | £850 | 8 | 8 |

Audited and found correct.

C. J. RYLAND & CO.

* These amounts should be reduced by £22 17s. 10d. : miscellaneous printing expenses to this amount, due by the *Journal*, were paid from the Society's account, The sum is included in the £261 2s. 4d. shewn as "Paid to Arrowsmith" in the *Journal* balance-sheet.

### BRISTOL MEDICO-CHIRURGICAL JOURNAL, 1949

### EXPENDITURE

| | £ | s. | d. | £ | s. | d. | £ | s. | d. |
|---|---|---|---|---|---|---|---|---|---|
| Arrowsmith, Printing: | | | | | | | | | |
| January, 1949 .. .. .. .. .. | | | | | | | 102 | 19 | 11 |
| April .. .. .. .. .. .. | | | | | | | 112 | 12 | 4 |
| July .. .. .. .. .. .. | | | | | | | 97 | 8 | 8 |
| October (estimate) .. .. .. | | | | | | | 152 | 11 | 4 |
| | | | | | | | £465 | 12 | 3 |
| 1948: | | | | | | | | | |
| Autumn | 56 | 0 | 7 | | | | | | |
| Winter .. | 54 | 8 | 5 | | | | | | |
| | | | | 110 | 9 | 0 | | | |
| Estimates: | | | | | | | | | |
| Autumn | 60 | 7 | 4 | | | | | | |
| Winter .. | 50 | 0 | 0 | | | | | | |
| | | | | 110 | 7 | 4 | | | |
| *Add* for 1948 excess over estimate .. | | | | | | | | 1 | 8 |
| Total .. .. .. .. .. .. | | | | | | | 465 | 13 | 11 |
| Postages and Sundries .. .. .. | | | | | | | | 4 | 0 |
| Audit Fee .. .. .. .. .. .. | | | | | | | 3 | 3 | 0 |
| | | | | | | | 469 | 0 | 11 |
| Surplus for year .. .. .. .. | | | | | | | 174 | 14 | 4 |
| | | | | | | | £643 | 15 | 3 |

### RECEIPTS

| | £ | s. | d. | £ | s. | d. |
|---|---|---|---|---|---|---|
| Subscriptions: | | | | | | |
| Members of Bristol M.-Chir. Soc. | | | | 380 | 0 | 0 |
| ,, Torquay M.-Chir. Soc. | | | | 15 | 10 | 0 |
| Non-members .. .. .. .. .. | | | | 44 | 14 | 6 |
| | | | | 440 | 4 | 6 |
| Advertisements: | | | | | | |
| January .. .. | 50 | 7 | 0 | | | |
| April .. .. .. | 53 | 3 | 9 | | | |
| July .. .. .. | 51 | 0 | 6 | | | |
| October (est.) .. | 48 | 19 | 6 | | | |
| | | | | 203 | 10 | 9 |
| | | | | £643 | 15 | 3 |

### LIABILITIES

| | £ | s. | d. | £ | s. | d. | £ | s. | d. |
|---|---|---|---|---|---|---|---|---|---|
| Creditors: Arrowsmith: | | | | | | | | | |
| September, 1948 | | | | 371 | 9 | 8 | | | |
| ,, 1949 .. | | | | 465 | 13 | 11 | | | |
| | | | | 837 | 3 | 7 | | | |
| *Deduct:* | | | | | | | | | |
| Paid, 1948 | 261 | 2 | 4 | | | | | | |
| Adverts | 203 | 10 | 9 | | | | | | |
| | | | | 464 | 13 | 1 | | | |
| | | | | | | | 372 | 10 | 6 |
| Auditors .. .. .. .. .. .. | | | | | | | 3 | 3 | 0 |
| | | | | | | | £375 | 13 | 6 |

### ASSETS

| | £ | s. | d. | £ | s. | d. |
|---|---|---|---|---|---|---|
| Cash at Bank .. .. .. .. .. | | | | 101 | 8 | 11 |
| ,, due from Soc. (inc. in £380 above) .. .. .. .. .. | | | | 179 | 2 | 2 |
| Deficit, Sept., 1948 | £269 | 16 | 9 | | | |
| *Less* Surplus for year .. | 174 | 14 | 4 | | | |
| | | | | 95 | 2 | 5 |
| | | | | £375 | 13 | 6 |

### Torquay and District Medical Society

A meeting was held at the Torbay Hospital on Thursday, March 9th, 1950, and a large body of local General Practitioners were addressed by Dr. Wilfrid Sheldon on "**Some Recent Advances in Paediatrics**". (P. 56.)

### British Medical Association

### Bath, Bristol and Somerset Branch

Wednesday, February 22nd, 1950, at the Royal United Hospital, Bath. Professor Robert Cruickshank on "**The Family Doctor as an Epidemiologist**".

Thursday, March 9th, 1950, at the Musgrove Park Hospital, Taunton. Dr. C. R. Gibson on "**Venus, Bacchus and Crime**".

### British Medical Association

### Bristol Division

Wednesday, February 15th, 1950. General Meeting of the Division in the Main Physics Lecture Theatre, University of Bristol.

